# Antimicrobial resistance and *mcr-1* gene in *Escherichia coli* isolated from poultry samples submitted to a bacteriology laboratory in South Africa

**DOI:** 10.14202/vetworld.2021.2662-2669

**Published:** 2021-10-20

**Authors:** Ibrahim Z. Hassan, Buks Wandrag, Johan J. Gouws, Daniel N. Qekwana, Vinny Naidoo

**Affiliations:** 1 Department of Paraclinical Sciences, Veterinary Pharmacology/Toxicology Section, Faculty of Veterinary Science, University of Pretoria, Pretoria, South Africa; 2 Department of Production Animal Studies, Faculty of Veterinary Science, University of Pretoria, Pretoria, South Africa; 3 Department of Veterinary Tropical Diseases, Faculty of Veterinary Science, University of Pretoria, Pretoria, South Africa; 4 Department of Paraclinical Sciences, Veterinary Public Health Section, Faculty of Veterinary Science, University of Pretoria, Pretoria, South Africa.

**Keywords:** antimicrobial resistance, colistin, *Escherichia coli*, mobilized colistin resistance-1, poultry

## Abstract

**Background and Aim:**

Antimicrobial resistance (AMR) and recently mobilized colistin resistance (*mcr-1*) associated colistin resistance among *Escherichia coli* isolates have been attributed to the overuse of antimicrobials in livestock production. *E. coli* remains an important pathogen, often associated with mortality and low carcass weight in poultry medicine; therefore, the need to use antimicrobials is common. The study aimed to determine the AMR profile and presence of *mcr-1* and *mcr-2* genes in avian pathogenic *E. coli* from poultry samples tested at a bacteriology laboratory for routine diagnosis. This is a first step in understanding the effectiveness of mitigation strategies.

**Materials and Methods:**

Fifty *E. coli* strains were assessed for resistance against ten antimicrobial drugs using broth microdilution. All isolates with a colistin minimum inhibitory concentration (MIC) of 2 μg/mL were analyzed for the presence of *mcr-1* and *mcr-2* genes by employing the polymerase chain reaction. For each isolate, the following farm information was obtained: farm location, type of farm, and on-farm use of colistin.

**Results:**

Sixty-eight percent of the strains were resistant to at least one antimicrobial; 44% were multiple drug-resistant (MDR). Most *E. coli* isolates were resistant to doxycycline (44%), trimethoprim-sulfamethoxazole (38%), ampicillin (32%), and enrofloxacin (32%). None of the *E. coli* strains was resistant to colistin sulfate (MIC_90_ of 2 μg/mL). Only one *E. coli* isolate held the *mcr-1* gene; none carried the *mcr-2* gene.

**Conclusion:**

Resistance among *E. coli* isolates in this study was fairly high. Resistance to commonly used antimicrobials was observed, such as doxycycline, trimethoprim-sulfamethoxazole, and enrofloxacin. Only a single *E. coli* strain carried the *mcr-1* gene, suggesting that *mcr-1* and *mcr-2* genes are common among isolates in this study. The prevalence of AMR, however, suggests that farmers must implement standard biosecurity measures to reduce *E. coli* burden, and antimicrobial use to prolong the efficacy life span of some of these drugs.

## Introduction

Antimicrobial resistance (AMR) is a global threat that has largely been the result of extensive misuse and overuse of antimicrobials for human and animal healthcare [1,2]. In food-producing animals, this inappropriate use of antimicrobial agents for growth promotion was described as a major driving factor [3,4]. This is exemplified by the recent emergence of a mobilized colistin resistance (mcr) mechanism, which has now spread globally attributable to veterinary colistin use [5-13]. To date, up to 10 diverse *mcr* genes with several variant types have been identified [5,6,8,10-13]. This emergence and AMR spread are linked to increased mortality and morbidity within significant clinical outcomes [1,2,14]. To discuss these concerns and maintain global access to therapeutically efficacious antimicrobials, the World Health Organization, Food and Agriculture Organization of the United Nations, the World Organization for Animal Health (Office International des Epizooties, and other stakeholders formulated a global action plan (GAP) as a way forward [4].

GAP aims to decrease selection pressure exerted by antibacterial agents on the emergence and spread of resistant bacteria [15]. To achieve this, concerted efforts are made toward collating and monitoring data on AMR. The outcome is expected to guide the selective use of antimicrobials to reduce global consumption and level of AMR [16,17].

Following GAP, this study was designed to collate data on AMR. This served to evaluate the effectiveness of one specific mitigation strategy, after the South African Veterinary Council, restricted use of colistin to critical cases shown to be only responsive to the drug, due to its global significance as one of the last resort treatment agents of human multiple drug-resistant (MDR) infections [18]. A constraint perceived with this restriction, was that the baseline for resistance was not properly established, nor were strategies implemented to monitor the effect of the same restriction. While only a few articles described the situation with the veterinary emergence of an mcr gene (*mcr-1*) in South Africa [7,9,19,20], these articles are not without shortcomings. The article by Theobald *et al*. [19] reports high resistance (>10% when intermediate and resistance strains are combined) in 2015 and did not report their minimum inhibitory concentration (MICs), whereas the study by Perreten *et al*. [7] did report their MIC, was limited to six farms. Most importantly, neither of these articles revealed if colistin was in use on monitoring the farms, complicating the process of linking drug use to resistance development.

Besides colistin, farmers have routinely employed additional antimicrobials therapeutically and prophylactically to manage animal diseases, reducing losses while maintaining productivity [12,21-23]. Avian colibacillosis caused by pathogenic *Escherichia coli* remains one of the most commonly encountered diseases of chickens in this regard [24,25]. The disease is associated with slowed growth rate (10%), mortality, and low carcass quality with 13% weight loss at slaughter [21,25-30]. *E. coli* is regarded as a major cause of food-borne diseases in humans [31]; therefore, the potential risk of resistant bacteria transmitted from animals to humans cannot be underestimated. More so that some Avian Pathogenic *E*. *coli* strains appear to be phylogenetically closely related to some Human Extra-Intestinal Pathogenic *E. coli* strains; they both share several common virulence factors [32-34].

Provided that only two studies demonstrated the presence of the *mcr-1* gene in the South Africa livestock industry, this study aimed to investigate the antimicrobial susceptibility profile and presence of *mcr-*1 and *mcr*-2 genes in *E. coli*. These were isolated from poultry samples submitted to a bacteriology laboratory in 2016, in the presence of a history of the use or non-use of colistin. Evaluating the AMR pattern of *E. coli* will provide valuable information, which should guide veterinary prescribing decisions. In addition, information on the *mcr* gene in *E. coli* poultry isolates will clarify the role of animals as reservoirs of AMR genes with the potential for transfer to other organisms [35]. Finally, the information forms the basis for future surveillance in determining the effectiveness of colistin control strategies.

## Materials and Methods

### Ethical approval

Ethical approval for this study was obtained from the University of Pretoria Animal Ethics Committee (V098-17).

### Study period and location

Samples were collected during the months of July and August 2016. The samples were processed at the Bacteriology Laboratory, Department of Veterinary Tropical Diseases, Faculty of Veterinary Science, University of Pretoria, South Africa.

### Study population and sampling

The study used 50 sequential *E. coli* isolates from clinical poultry (*Gallus gallus*) samples submitted to the bacteriology laboratory of the Faculty of Veterinary Science, the University of Pretoria, for diagnosis. For each isolate, information on farm location, type of farm, and on-farm use of colistin was recorded. As a result, the sampling was completely random.

### Isolation of *E. coli*

*E. coli* bacteria were isolated from samples submitted to the laboratory by employing standard procedures [36,37]. Organisms were grown on MacConkey agar and blood agar (Thermo Fischer Scientific, South Africa). The resulting colonies were subsequently subjected to biochemical tests. Presumptive colonies were confirmed by employing the API 10 S test (bioMérieux, France). The resulting four number digits were entered into the API Web^®^ database to categorize the organisms.

### Antimicrobial susceptibility testing

The antimicrobial susceptibility of all isolates was determined using the broth microdilution technique based on the Clinical and Laboratory Standards Institute (CLSI) guidelines [38]. The antimicrobial drug compounds tested included colistin, ampicillin, fosfomycin, enrofloxacin, doxycycline, neomycin, sulfa-trimethoprim, kanamycin, ceftiofur, and cefoxitin. The lowest antimicrobial concentration with no evidence of growth is regarded as the MIC. For quality control, *E. coli* ATCC 25922 was used. Interpretation of antimicrobial susceptibility was undertaken as described in the 2018 CLSI guideline for all drug compounds except colistin. The EUCAST guideline of 2018 was used to interpret colistin susceptibility [39]. *E. coli*, resistant to more than two antimicrobials, was categorized as MDR.

### Polymerase chain reaction (PCR) and gel electrophoresis for *mcr-1* and *mcr-2* genes

The colistin susceptible *E. coli* strains harboring the *mcr-1* gene have been identified several times from farms with no history of previous drug use [40,41]. All eight strains obtained from farms with no history of the previous colistin use, and with the maximum colistin MIC (2 μg/mL) for this study were, therefore, recruited for PCR. Besides the limited financial resources, part of the rationale for testing only *mcr*-*1* and *mcr*-*2* genes was that these genes were the most commonly associated with colistin susceptible strains, and most prevalent globally [42]. Deoxyribonucleic acid (DNA) was extracted from the strains using the boiling method and a multiplex PCR was undertaken. A Qiagen multiplex PCR kit (Qiagen) was used with the primer sequences *mcr*1-F 5′ CGGTCAGTCCGTTTGTTC 3′, *mcr*1-R 5′ CTTGGTCGGTCTGTAGGG 3′, *mcr*2-F 5’ TGGTACAGCCCCTTTATT 3’, and *mcr*2-R 5’GCTTGAGATTGGGTTATGA 3’[5,6]. The initial DNA denaturation step was undertaken at 94°C for 15 min. This was followed by 25 cycles of sequence amplification steps, such as denaturation (at 94°C for 30 s), annealing (at 58°C for 90 s), and extension (at 72°C for 60 s). The final elongation step was at 72°C for 10 min. The resulting amplicons were subjected to agarose gel electrophoresis using 1.5% gel in 1×TBE with ethidium bromide. The gels ran for 90 min at 90 volts before visualizing the bands [43].

### Sequencing of *mcr-1* gene amplicon

An amplicon was sent in for *mcr-1* gene sequencing, confirming that the proper gene was amplified. A Sanger sequencing platform was employed for the analysis through a commercial laboratory. The resulting nucleotide sequence was blasted on the NCBI database. The sequence was also aligned with other *mcr-1* genes and variants sequences from the NCBI database, employing MEGA X software [44]. A phylogenetic tree was drawn to demonstrate the evolutionary relationship among the genes. Twenty diverse nucleotide sequences were compared.

### Statistical analysis

AMR profiles of isolates and information on-farm use of colistin were coded and entered on Statistical Package for the Social Sciences (SPSS) (IBM SPSS Statistics for Windows, Version 26.0. IBM Corp., NY, USA) for descriptive analysis. The proportions of AMR/MDR concerning provinces and drugs were evaluated, and their 95% CI were calculated. Association was assessed by employing the Chi-square test or Fischer’s exact probability, where more than 20% of the cells had expected frequencies <5. Proportions of strains concerning farm use of colistin were determined. The MIC, MIC_50_, and MIC_90_ for colistin are also presented.

## Results

### Isolates

Forty-six percent (23/50) of the isolates originated from the North West Province; 40% (20/50) from Gauteng; and 14% (7/50) from Mpumalanga. Concerning the animal type, 50% (25/50) of the isolates were obtained from broilers, whereas the remaining half came from layers 50% (25/50).

### Antimicrobial susceptibility

Sixty-eight percent (34/50) of the *E. coli* isolates were resistant to at least one antimicrobial assessed. *E. coli* isolates demonstrated high proportions of resistance to doxycycline (44%, 22/50), trimethoprim-sulfamethoxazole (38%, 16/50), ampicillin (32%, 16/50), and enrofloxacin (32% (16/50). Low proportions of resistance were observed to ceftiofur (10%, 5/50) and neomycin (6%, 3/50). None of the *E. coli* strains was resistant to colistin sulfate (Table-1). Isolates from the North West demonstrated the highest proportion of AMR to drugs tested (Table-1). The next was Mpumalanga with Gauteng indicating the least level of AMR (p<0.05).

**Table-1:** Proportions of AMR in clinical Avian *E. coli* isolates, obtained from South Africa in 2016.

Variable	Percentage AMR	95% CI		Percentage MDR	95% CI	
	
		Lower	Upper		Lower	Upper
Drug compounds						
Colistin sulfate	0 (0/50)	0.00	7.1	0 (0/22)	0.00	15.4
Ampicillin	32 (16/50)	19.5	46.7	68.2 (15/22)	45.1	86.1
Fosfomycin	14 (7/50)	5.8	26.7	27.3 (6/22)	10.7	50.2
Enrofloxacin	32 (16/50)	19.5	46.7	59.1 (13/22)	36.4	79.3
Doxycycline	44 (22/50)	30.0	58.7	77.3 (17/22)	54.6	92.2
Neomycin	6 (3/50)	1.3	16.5	13.6 (3/22)	2.9	34.9
Sulfamethoxazole/trimethoprim	38 (19/50)	24.7	52.8	63.6 (14/22)	40.7	82.8
Kanamycin	8 (4/50)	2.2	19.2	18.2 (4/22)	5.2	40.3
Ceftiofur	10 (5/50)	3.3	21.8	22.7 (5/22)	7.8	45.4
Cefoxitin	20 (10/50)	10.0	33.7	36.4 (8/22)	17.2	59.3
Provinces						
Mpumalanga	71.4 (5/7)	29.0	96.3	22.7 (5/22)	7.8	45.4
Gauteng	50 (10/20)	27.2	72.8	31.8 (7/22)	13.9	54.9
North West	82.6 (19/23)	61.2	95.0	45.5 (10/22)	24.4	67.8

AMR=Antimicrobial resistance, *E. coli=Escherichia coli*, MDR=Multiple drug-resistant

MDR was observed in 44% (22/50) of isolates (Table-1) with MDR-*E. coli* mainly resistant to ampicillin (68.2%, 15/22), enrofloxacin (59.1%, 13/22), doxycycline (77.3%, 17/22), and trimethoprim-sulfamethoxazole (63.6%, 14/22). No significant difference was observed (p=0.422) in the proportions of isolates that displayed MDR among the three provinces.

No clear pattern in strains display of MDR was observed; however, resistance toward ampicillin, doxycycline, trimethoprim/sulfamethoxazole, and enrofloxacin, were common. Importantly, one of the isolates obtained was resistant to all antimicrobials assessed except for colistin, neomycin, and cefoxitin (Table-2).

**Table-2:** Multiple drug resistance patterns of clinical Avian *E. coli* strains obtained from South Africa in 2016.

MDR pattern	Number of isolates	Resistance pattern
1	1	Enro, Dox and Sulf/Trim
2	2	Amp, Dox and Sulf/Trim
3	1	Fos, Dox and Sulf/Trim
4	1	Amp, Enro and Sulf/Trim
5	2	Amp, Enro and Dox
6	2	Fos, Enro and Sulf/Trim
7	2	Amp, Enro, Dox and Sulf/Trim
8	1	Fos, Enro, Dox and Cefx
9	1	Enro, Dox, Neo and Kan
10	1	Enro, Dox, Sulf/Trim and Cefx
11	1	Amp, Neo, Ceft and Cefx
12	1	Amp, Dox, Ceft and Cefx
13	1	Amp, Enro, Dox and Cefx
14	1	Amp, Sulf/Trim, Ceft and Cefx
15	1	Amp, Fos, Dox, Sulf/Trim and Kan
16	1	Amp, Dox, Neo, Ceft and Cefx
17	1	Amp, Dox, Sulf/Trim, Kan and Cefx
18	1	Amp, Fos, Enro, Dox, Sulf/Trim, Kan and Ceft

Amp=Ampicillin; Fos=Fosfomycin; Enro=Enrofloxacin; Dox=Doxycycline; Sulf/Trim=Sulfamethoxazole Trimethoprim; Neo=Neomycin; Kan=Kanamycin; Ceft=Ceftiofur; Cefx=Cefoxitin. *E. coli=Escherichia coli*, MDR=Multiple drug resistant

### Colistin MIC and farm use of colistin

Of the 50 strains tested, 84% (42/50) were obtained from farms with no history of colistin use. The remaining 6% (3/50) and 10% (5/50) originated from farms that used the drug previously (6-18 weeks ago) and those who were currently using the drug, respectively. The colistin MICs ranged from 0.125 μg/mL to 2 μg/mL, with an overall MIC_90_ of 2 μg/mL (Table-3).

**Table-3:** Distribution pattern of Colistin MIC (µg/mL) in clinical Avian *E. coli* isolates obtained from South Africa in 2016.

Farm use of colistin	Average MIC	N	Mode	Max MIC	Min MIC	MIC SD	MIC50	MIC90
Never	0.88	42	0.5	2.00	0.125	0.62	0.5	2
Previous	0.42	3	0.5	0.50	0.250	0.14	0.5	0.5
Recent	0.28	5	0.25	0.50	0.125	0.14	0.25	0.5
Total	0.79	50	0.5	2.00	0.125	0.60	0.5	2

MIC=Minimum inhibitory concentration; N=Number of isolates; MIC_50_=Minimum inhibitory concentration which inhibits 50% of the isolates; MIC_90_=Minimum inhibitory concentration which inhibits 90% of the isolates; MIC SD=Standard deviation of Minimum inhibitory concentrations; Min MIC=Least MIC; Max MIC=Maximum MIC. *E. coli=Escherichia coli*

### PCR and gel electrophoresis for *mcr-1* and *mcr-2* genes

Of all the eight assessed isolates, the PCR demonstrated the presence of the *mcr-1* gene in only one of the colistin susceptible *E. coli* strains. In contrast, no *mcr-2* gene was detected following the PCR.

### Sequencing of *mcr-1* gene amplicon

**Figure-1:**
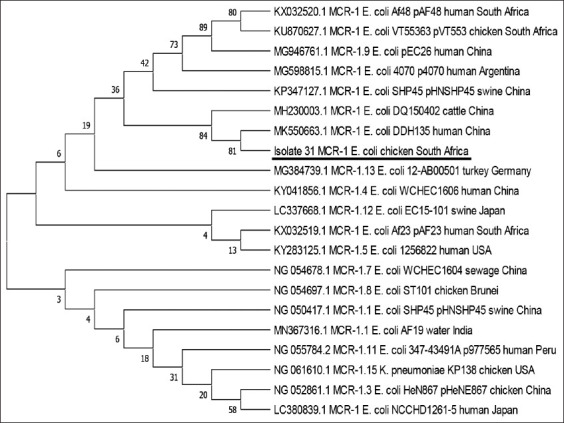
Evolutionary tree of mobilized colistin resistance (*mcr)-1* gene sequence obtained from the present study in comparison with sequences of other *mcr-1* variants.

Gene sequencing confirmed the isolate to be *mcr-1* positive. A blast of the nucleotide sequence on the NCBI database revealed 97% query coverage, aligning well with other *mcr-1* genes and variants sequences in the database. Phylogenetic analysis with MEGA X software clustered the sequence with sequences (MH230003.1, MK550663.1) from China (Figure-1).

The evolutionary history was inferred by employing the maximum likelihood method and Tamura-Nei model [45]. The bootstrap consensus tree inferred from 500 replicates [46] is taken to represent the evolutionary history of the taxa analyzed [46]. Branches corresponding to partitions reproduced in <50% bootstrap replicates are collapsed. The percentage of replicate trees where the associated taxa clustered in the bootstrap test (500 replicates) are indicated next to the branches [46]. The initial tree(s) for the heuristic search were automatically obtained by applying Neighbor-Join and BioNJ algorithms to a matrix of pairwise distances. These were estimated with the Maximum Composite Likelihood approach. The topology with a superior log-likelihood value was then selected. This analysis involved 21 nucleotide sequences. There were 66834 positions in the final dataset. Evolutionary analyses were conducted in MEGA X [44].

## Discussion

This study was undertaken to determine the AMR and presence of *mcr-1* and *mcr*-*2* genes in *E. coli* isolated from clinical poultry samples. The study investigated the relationship between colistin use and colistin resistance. The proportion of *E. coli* resistant to at least one antimicrobial tested (68%) was similar to that of the SANVAD report (67%) of 2007 [35]. This raises a perplexing question as to whether the mitigation strategies on prudent use of antimicrobials, in line with Acts (i.e., Act 36/1947 and Act 101/1965) regulating use were effective [47]. It was expected that the proportions of AMR-*E. coli* strains would gradually decrease. The possible reasons could be the differences in antimicrobial drugs tested. The SANVAD report reflects aggregated data obtained from multiple animal species, including chicken.

### Colistin resistance among *E. coli* isolates

The results suggest that the prevalence of colistin resistance in clinical poultry isolates in this study is low, evident from the inability of the present study to detect any form of colistin resistance and with a colistin MIC_90_ of 2 μg/mL. This observation may relate to the legislation guiding the veterinary use of the drug in South Africa, restricting its prescription for veterinarians alone, which invariably limits unchecked access to the drug [19,48]. This low level of colistin use is reflected in the findings of this study as most (84%) farms never used the antimicrobial before. Of interest is that even on farms where colistin was used (16%), strains were considered wild types since their MICs were below the recommended breakpoint. These findings suggest that the colistin use on these farms had minimal selection pressure for colistin resistance among *E. coli* isolates.

### Presence of *mcr-1* gene among *E. coli* isolates

Earlier reports in South Africa demonstrated a rising level of colistin resistance, with a 95% *mcr-1* gene carriage among resistant strains [7]. This identification of the *mcr-1* gene is not peculiar to South Africa [14,49-56]. In China, where *mcr-1* associated colistin resistance was discovered [5], one of the reasons identified was that this could be related to low dose use of colistin for growth promotions [57,58]. In contrast, studies indicated that the levels of resistance in Europe were lower attributable to the drug being used sparingly at higher therapeutic doses [57,58].

In the present study, the *mcr-1* gene was noted in one colistin susceptible *E. coli* organism from a farm with no history of the previous colistin use. While this is not the first reported case [40,41,59], it emphasizes the importance of other factors contributing to AMR. Several studies traced back AMR strains in poultry populations under no selective pressure to breeding farms [60-62]. Once introduced, some of these strains could persist in the farm environment even after intensive cleaning and disinfection routines were undertaken [63-65]. Given that this is the first study in South Africa to demonstrate the presence of *mcr-1* gene in a susceptible strain, it is possible that the *E. coli* with the *mcr-1* gene were brought in mechanically to the farm. A BLAST search of its sequence on the NCBI database revealed extremely high similarities with sequences reported in Asia and South America, especially the human strain (MH230003.1, MK550663.1). These similarities were also reflected on the phylogenetic tree generated, clustering the gene sequence with sequences from the regions mentioned.

### Fluoroquinolones resistance among *E. coli* isolates

The reservation of colistin may leave farmers relying on enrofloxacin to manage colibacillosis. This is problematic as it may increase resistance. In this study, a higher proportion of enrofloxacin resistance (32%) among *E. coli* was observed compared to the 21.1% reported by Theobald *et al*. [19] in 2015. This could, therefore, be attributed to selective pressure from increased poultry industry use because of the drug’s observed therapeutic efficacy in the management of avian colibacillosis in preceding years. The number of fluoroquinolones imported to South Africa for veterinary use as reflected in the 2014 and 2015 data, had increased by a 10% margin from 6.3 tons [66]. This may also have implications for medical use as the human analog of enrofloxacin (such as ciprofloxacin) is a metabolite of the former [67,68]. An increase in its consumption in poultry may coselect for resistance in both with devastating consequences, especially in the treatment of community-acquired Enterobacteriaceae infections where ciprofloxacin is indicated as a first-line drug [69]. In 2005, the USA banned the veterinary use of enrofloxacin and sparfloxacin in poultry medicine attributable to the rising human fluoroquinolone-resistant campylobacteriosis cases [70,71].

### Multiple drug-resistant *E. coli* isolates

One disturbing finding in this study is the MDR pattern displayed by organisms involving tetracyclines, penicillin, and sulfonamides. These drugs are commonly used in the poultry industry, suggesting that the MDR pattern could be linked to the high consumption of these drugs in veterinary medicine. Even though relative to human medicine, veterinary consumption is a quarter of all antimicrobials imported into the country. The aforementioned classes of drugs are among the most imported for veterinary use despite being considered medically important. Coincidentally, they also represent the most sold veterinary antibiotic classes in the United Kingdom [72]. However, considering that South Africa’s consumption of veterinary antimicrobials is much higher than most EU countries UK inclusive, despite the livestock industry in the UK being more than twice the size of South Africa, the fairly high level of MDR (44%) identified in this study may be as a result of drug overuse. It also emphasizes the need for adequate measures to prolong the efficacy life span of some of these drugs as effort should be directed at harmonizing acts regulating the veterinary use of antimicrobials to limit uncontrolled access to farmers.

### Limitations of the study

This study used isolated samples submitted to one laboratory; therefore, the findings of this study cannot be generalized to all chickens in South Africa. This study focused on clinical cases from commercial farms. The results may, therefore, be skewed to reflect farms with a history of antimicrobial use, complicating cases. This study employed a 2 μg/mL criterion for recruiting strains for *mcr-1* and *mcr*-*2* genes testing; however, several studies demonstrated the presence of the *mcr-1* gene in bacterial organisms with far lesser colistin MICs than 2 μg/mL used in this study [41,73,74]. The prevalence of *mcr* genes could be underestimated since not all isolates were tested.

## Conclusion

Detecting the *mcr-1* gene in a colistin susceptible strain was significant though the outcome of this study demonstrates that colistin resistance was not common among *E. coli* isolates from poultry. It is, therefore, recommended that all relevant clinical bacterial isolates undergo screening for the *mcr-1* gene to mitigate any form of gene propagation. The presence of MDR-*E. coli*, as demonstrated here, could be rendering drug use ineffective in poultry medicine, with a future negative impact on production and animal welfare. As a result, stricter measures need to be implemented to prevent uncontrolled access to antimicrobials.

## Authors’ Contributions

VN: Conceptualization. IZH: Formal analysis. VN: Funding. JJG and BW: Investigation. VN and IZH: Project administration. BW: Resources. VN and DNQ: Supervision. JJG: Validation. IZH and VN: Writing – original draft. IZH, VN, and DNQ: Writing – review and editing. All authors read, revised, and approved the final manuscript.
